# Description of *Ektaphelenchus koreanus* n. sp. (Nematoda: Ektaphelenchidae) with morphometrical notes on the *Ektaphelenchus* species

**DOI:** 10.21307/jofnem-2019-049

**Published:** 2019-07-29

**Authors:** Jianfeng Gu, Munawar Maria, Yiwu Fang, Lele Liu

**Affiliations:** 1 Technical Center of Ningbo Customs, 8 Huikang Road, Ningbo 315000, Zhejiang, P.R. China; 2 Laboratory of Plant Nematology, Institute of Biotechnology, College of Agriculture and Biotechnology, Zhejiang University, Hangzhou, 310058, Zhejiang, P.R. China

**Keywords:** Distribution, New species, Korea, Intercepted, Morphometry, Morphology, DNA sequencing, Taxonomy

## Abstract

*Ektaphelenchus koreanus* n. sp. is isolated from *Pinus* packaging wood from Korea in Ningbo customs. The new species can be characterized by having four incisures in the lateral field, the excretory pore located posterior to nerve ring, female devoid of vulval flap, and having long post-vulval intestinal sac (55–106 μm), tail with a finely rounded terminus. Six caudal papillae in male and spicule short (12.7–13.7 μm), having broad squared to rounded condylus, triangular rounded rostrum, cucullus absent. Morphologically the species is most similar with *E. berbericus*, *E. joyceae*, *E. oleae*, *E. ibericus*, and *E. taiwanensis* but it can be differentiated by plenty of morphometrical and morphological characters. In addition, a morphometry table of *Ektaphelenchus* is also presented.

Most of *Ektaphelenchus* (Fuchs, 1937) species were found in association with beetles (Rühm, 1956; Massey, 1974). The word *Ektaphelenchus* derived from the Greek word *ektos*=outside and *apheles*=smooth, *enchus*=spear (Hunt, 1993). Currently, the genus contains 28 species that have been reported from Europe, North America, Russia, Iran, India, and China (Yang, 1985; Hunt, 1993; Alvani et al., 2016; Miraeiz et al., 2017). Three *Ektaphelenchus* were detected in the quarantine samples from Korea, Taiwan, and Spain examined at Ningbo customs, China, indicating that the known distribution range has increased (Gu et al., 2013a, 2013b). Unlike *Bursaphelenchus* and *Aphelenchoides* species, Ektaphenchid nematodes are not qualified as quarantine pests but their presence in the examined sample complicates the phytosanitary inspections. Therefore, the detection of another *Ektaphelenchus* species at Ningbo customs leads us to perform morphological, morphometrical, and molecular studies which revealed the status of this species as a new species and it is described herein *E. koreanus* n. sp.

## Materials and methods

### Nematode isolation and morphological study

Sawn samples taken from logs were cut into small pieces *ca* 1 cm wide and 10 cm long. Nematodes were extracted by a modified Baermann funnel technique for 24 hr. For morphometric studies, the extracted individuals were killed by heat, fixed with FA 4:1, and processed via ethanol-glycerin dehydration, according to Seinhorst (1959), as modified by De Grisse (1969) and mounted in glycerin on slides. The measurements and light micrographs of nematodes were made using a Zeiss Imager Z1 microscope equipped with a Zeiss AxioCam MRm CCD camera.

### Molecular and phylogenetic analyses

DNA was extracted from single nematode into an Eppendorf tube. The nematode was crushed and the sample was processed for extraction as described by Zheng et al. (2003). The ITS region was amplified with the forward primer F194 (Ferris et al., 1993) and the reverse primer 5368r (Vrain, 1993). PCR products were separated on 1% agarose gels and visualized by staining with ethidium bromide. PCR products of sufficiently high quality were purified for cloning and sequencing by Majorbio, Shanghai, China. The sequences of the ITS region of *E. koreanus* n. sp. were compared with those of other *Ektaphelenchus* species available in GenBank using the BLAST homology search program. The selected sequences were aligned by MAFFT (Katoh and Standley, 2013) with default parameters. The alignments of sequences were manually edited and assembled in one data set by using AliView (Larsson, 2014). The best-fitted model of DNA evolution was obtained using jModelTest2 (Darriba et al., 2012) with the Akaike information criterion (AIC). The Bayesian tree was inferred using MrBayes 3.2.3 (Ronquist and Huelsenbeck, 2003) with four chains (three heated and one cold). Model parameters were unlinked and the overall rate was allowed to vary across partitions. The number of generations for the total analysis was set to 1×10^8^, with the chains sampled every 1,000 generations and the burn-in value set to 25%. The Markov chain Monte Carlo (MCMC) method within a Bayesian framework was used to estimate the posterior probabilities of the phylogenetic trees using the 50% majority rule (Larget and Simon, 1999). The consensus tree was selected to represent the phylogenetic relationships with branch length and support level and visualized using TreeGraph 2 (Stöver and Müller, 2010).

## Results

Systematics

*Ektaphelenchus koreanus* n. sp.

(Figs. 1–2).

**Figure 1:**
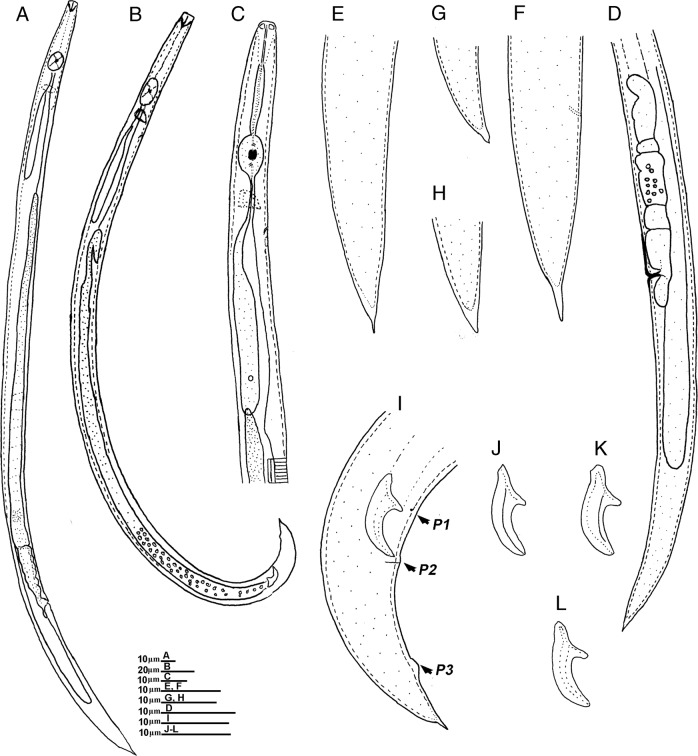
Line drawings of *Ektaphelenchus koreanus* n. sp. (A) female; (B) male; (C) head region; (D) posterior part of female; (E–H) female tail region; (I) lateral view of male tail; (J–L) spicules. (Scale bars = A–L = 10; B = 20 μm).

**Figure 2:**
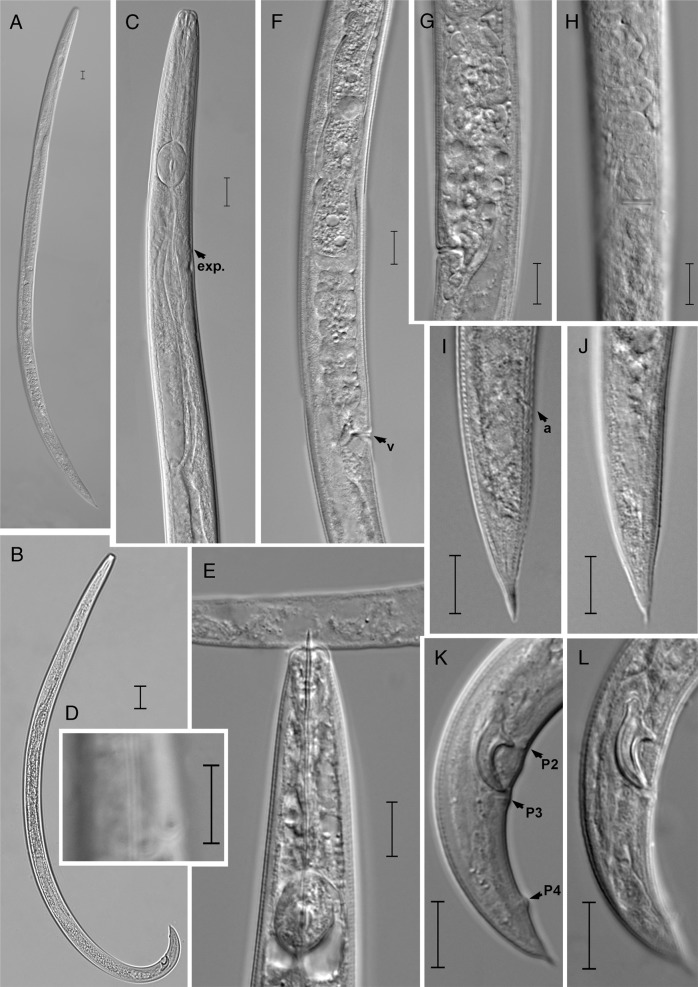
Light photomicrographs of *Ektaphelenchus koreanus* n. sp. (A) female; (B) male; (C) head region; (D) lateral region; (E) feeding on *Aphelenchoides* sp.; (F, G) vulval region (lateral view); (H) vulval region (ventral view); (I, J) female tail; (K, L) Male tail. (Scale bars = 10 μm).

### Measurements

Measurements of the new species are given in Table 1.

**Table 1.** Morphometrics data for *Ektaphelenchus koreanus* n. sp. All measurements are in µm and in the form of mean ± s.d. (range).

	Female		Male
Characters	Holotype	Paratypes	Paratypes
n	–	20	8
L	631	585 ± 68.2 (474–705)	475 ± 20.0 (448–517)
a	32.3	33.1 ± 1.7 (30.2–36.4)	32.5 ± 2.5 (27.2–35.4)
b	10.5	9.9 ± 0.8 (8.2–10.9)	8.1 ± 0.6 (7.2–8.7)
b′	4.2	4.0 ± 0.4 (3.2–4.6)	3.5 ± 0.2 (3.3–4.0)
c	–	–	16.5 ± 0.9 (15.2–18.2)
c′	–	–	2.6 ± 0.2 (2.3–3.0)
V or T	77.9	77.3 ± 0.7 (75.7–78.1)	31.3 ± 3.4 (27.3–38.1)
Max body diam	19.5	17.8 ± 2.4 (14.0–21.8)	14.7 ± 1.2 (13.4–16.7)
Lip diam	8.2	6.8 ± 0.9 (5.5–8.2)	6.6 ± 0.2 (6.3–7.0)
Lip height	3.9	3.2 ± 0.6 (2.3–4.3)	2.6 ± 0.2 (2.2–2.9)
Stylet length	15.8	14.0 ± 0.8 (12.4–16.4)	13.5 ± 0.9 (12.4–14.9)
Median bulb length	16.2	15.9 ± 0.8 (13.6–17.1)	14.8 ± 0.7 (14.0–15.9)
Median bulb diam	10.8	10.3 ± 0.9 (8.2–11.7)	9.6 ± 0.7 (8.5–11.0)
Median bulb length/diam	1.5	1.6 ± 0.1 (1.4–1.8)	1.5 ± 0.0 (1.5–1.6)
Excretory pore position	89.6	86.7 ± 4.7 (77.1–93.5)	86.1 ± 5.5 (76.3–92.7)
Spicule(chord)	–	–	13.2 ± 0.3 (12.7–13.7)
Ovary or testis length	372	298 ± 66.7 (201–411)	148 ± 14.1 (130–171)
Post-uterine sac length	14.5	11.3 ± 2.1 (7.1–14.7)	–
Post vulval intestinal/blind sac	102	81.9 ± 16.4 (55.0–106.0)	–

Description.

### Female

On fixation, the body assumes slightly ventrally arcuate posture. The cuticle is finely annulated having four incisures in the lateral field. Labial region is hemispherical, weakly set off. Stylet is 12.4 to 16.4 μm long, divided into two parts without small basal swellings and conus occupying ca 40% of length. Procorpus is cylindrical, metacorpus (median bulb) is strongly developed, spherical with centrally valves. Dorsal pharyngeal gland orifice has opening into lumen of metacorpus *ca* about one metacorpal valve length anterior to metacorpal valve. Pharyngo-intestinal junction is located one metacorpal valve length posterior to metacorpus. Nerve ring *ca* is one metacorpal length posterior to metacorpus. Pharyngeal gland lobe is well developed, overlapping intestine dorsally. Excretory pore is located at the same level or slightly posterior to the nerve ring. Hemizonid is immediately posterior to excretory pore. Single ovary is ventrally located and is outstretched, developing oocytes arranged in 1 to 2 rows with several well-developed oocytes arranged in single row. Oviduct is short and connected with square-shaped spermatheca filled with sperm cells. Vagina is a straight, vulva a traverse slit, with hemispherical vulval lips. Post-uterine sac is small, 7.1 to 14.7 μm long. Anus indistinct. Rectum is poorly developed, indistinct in most of specimens, vestigial and presumably non-functional, and intestine in most specimens is apparently a blind sac. Tail is conical, tapering gradually to terminus forming a finely rounded tip, and in some individuals it is tapering suddenly before the tail terminus and appears like a mucron, 2 to 5 μm long.

### Male

Body is slender, cylindrical, strongly ventrally arcuate when heat-relaxed. Cuticle and anterior body region are similar to those of female. Single testis that is located on the left side of intestine and is outstretched, developing spermatocytes in one single column. Cloacal lips are not protruding. Spicules are paired, condylus is broad and rounded to triangular tip, rostrum is triangular with a rounded tip, capitulum is slight depressed near calomus-rostrum junction, and lamina is with a dorsal pronounced curvature till distal end. Distal end of spicule is smooth, and cucullus is absent. Three pairs of subventral papillae are present: one pair precloacal is located at the level of rostrum, one pair postcloacal is located just below the cloacal opening, and one pair is situated at posterior part of the tail. Tail is conoid, with a finely pointed terminus. Bursa is absent.

### Type host and locality

The type material was isolated from *Pinus* packaging wood imported from Korea on May, 2011 and inspected at Ningbo port, P.R. China.

### Type specimens

Holotype female, 5 male and 17 female paratypes (slide numbers 43968-1 to 43968-6) were deposited in the nematode collection of Ningbo Entry-Exit Inspection and Quarantine Bureau, China. Two paratype females and three paratype males (slide numbers 11557) were deposited in the Canadian National Collection of Nematodes, Ottawa, ON, Canada.

### Etymology

The species epithet is formed from the presumed country of origin.

### Differential diagnosis

The *Ektaphelenchus koreanus* n. sp. can be characterized by the lateral field with four lines, excretory pore located at the level or slightly posterior to nerve ring, spicules paired, broad condylus with rounded to triangular tip, rostrum triangular with a rounded tip, capitulum with slight depression near calomus-rostrum junction, the distal end of spicule smooth, cucullus absent, three pairs of caudal papillae present. Female tail conical that tapers gradually to terminus forming a finely rounded tip.

After a list of 24 valid species presented by Hunt (2008), four new *Ektaphelenchus* species were described. Unlike other Aphelenchids, no character scheme was proposed to identify the member species in this genus, several old descriptions are in different languages, hence it is slightly difficult to assign a true status.

**Figure 3:**
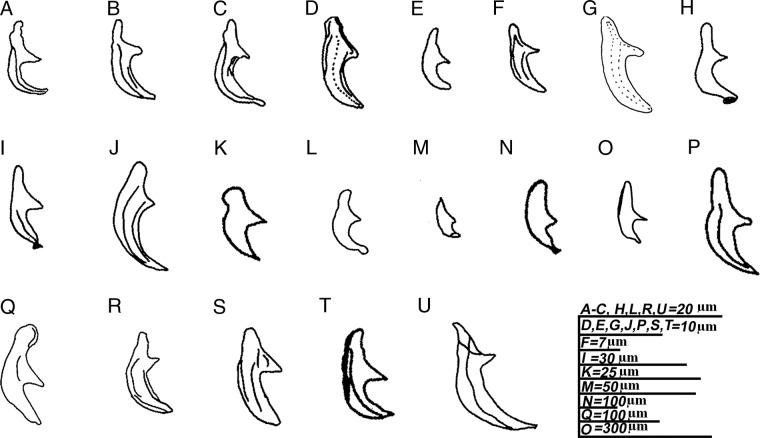
Spicule morphology of accessible *Ektaphelenchus* species. (A) = *E. betulae*; (B) = *E. dendroctoni*; (C) = *E. goffarti*; (D) = *E. ibericus*; (E) = *E. josephi*; (F) = *E. joyceae*; (G) = *E. koreanus* n. sp.; (H) = *E. macrobulbosus*; (I) = *E. obtusus*; (J) = *E. oleae*; (K) = *E. piniperdae*; (L) = *E. prolobos*; (M) = *E. propora*; (N) = *E. riograndensis*; (O) = *E. sandiaensis*; (P) = *E. scolyti*; (Q) = *E. skrjabini*; (R) = *E. stammeri*; (S) = *E. taiwanensis*; (T) = *E. tuerkorum*; (U) = *E. zwoelferi*. Scale bars (A–C, H, L, R, U, S = 20 µm; D, E, G, J, P, S, T = 10 µm; F = 7 µm; I = 30 µm; K = 25 µm; M = 50 µm; N = 100 µm; Q = 100 µm, O = 300 µm).

Lateral lines have been considered as a consistent character for species differentiation by several authors (Ryss et al., 2005; Braasch et al., 2009). The valid *Ektaphelenchus* species have been listed according to a number of incisures in Table 2, but many species were described without mentioning the lateral lines. Another diagnostic character is the spicule morphology (Kanzaki, 2006; Braasch et al., 2009; Maria et al., 2015), available species spicule drawn and presented in Fig. 3, but this characters cannot be used when the species are only described with female specimens. By examining all the literature on *Ektaphelenchus* species, we propose the female tail morphology as a prime character for species differentiation.

**Table 2.** Morphometrical values of *Ektaphelenchus* species.

Species		L	Stylet L	Spicule L	Vulva to tail terminus	a	b	c	V/T	Reference
					Species having 2 incisures in the lateral field					
*E. josephi*	Male	710	–	–	–	32	7	12		Massey (1974)
	Female	830–920	22	–	–	29.5–33	8–9	–	75–77	
*E. riograndensis*	Male	670–810	–	–	–	24–34	6.3–8	1.6–1.8	–	Massey (1964a, 1964b)
	Female	750–910	–	–	–	25–34	6.4–8.5	–	80	
	Male	620–650	21–22	20–22.5	–	26–31	7.1–8.1	2.5–2.6		Kaisa (2000)
	Female	630–780	22–23	–	–	25.2–31.5	6.8–9.5	–	79–81	
					Species having 3 incisures in the lateral field					
*E. joyceae*	Male	400–470	14–16	13–14	–	26–35	5–7	15–18	–	Kaisa, et al. (1995)
	Female	550–740	14–18	–		29–44	7–13	–	77–86	
*E. macrobulbosus*	Male	432–463	14–16	13	–	25.7–30.8	9.1–9.3	20.5–22.0	–	Rühm (1956)
	Female	518–576	16	–	93–112	24–28.7	9.9–10.8	5.1–5.6	80.5–82.1	
*E. obtusus*	Male	700	–	18.5	–	23	7	–	–	Massey (1956)
	Female	800	24		–	30	8	–	41–78	
	Male	968	–	22.5	–			20.6		Kanzaki et al. (2008) ^b^
	Female	648–881	24–30	–	–	25.9–33.3	6.4–9.8	–	71.8–79	Kanzaki et al. (2008) ^c^
*E. sandiaensis*	Male	620–640	–	16	–	32	8.0–8.5	16	–	Massey (1964a)
	Female	630–640	–	–	–	28–31	8.0–8.5	–	–	
*E. taiwanensis*	Male	474–540	11.9–15.7	11.3–13.1	–	26.6–31.9	6.3–8.6	15.2–18.6	–	Gu et al. (2013a)
	Female	482–661	12–16.9	–	–	26.8–31.1	7.2–9.6	–	76.7–79	
					Species having 4 incisures in the lateral field					
*E. ibericus*	Male	453	13.1	12.8	–	32.8	7.5	15.4	–	Gu et al. (2013b)
	Female	475–624	11.2–14.6	–	–	30.1–34.7	8–9.6	–	76–78	
*E. koreanus* n. sp.	Male	448–517	12.4–14.9	12.7–13	–	30.2–36.4	7.2–8.7	15.2–18.2	27.3–38.1	This study
	Female	474–705	12.4–16.4	–	–	27.2–35.4	8.2–10.9	–	75.7–78.1	
*E. olea*	Male	452–602	15–17	16–18	–	26.5–33	6.7–8	14–16.5	–	Miraeiz et al. (2017)
	Female	441–652	15–19	–	–	28–41	6.9–8.7	14.8–19	70–81.5	
					Species with unknown incisures in lateral field					
*E. betulae*	Male	600–675	17	15–17	–	34.3–38	6.6–7.4	21.5–22.5	–	Rühm (1956)
	Female	750–945	21	–	180–225	27–30	7.4–9.3	4.1–4.2	76–77	
*E. dendroctoni*	Male	720–810	22	18	–	29.4–30	7.3–7.4	18.9–20.2	–	Rühm (1956)
	Female	780–870	23	–	191–196	31.1–31.8	6.9–8.0	3.9–4.6	74–78	
*E. goffarti*	Male	442–485	16–18	13–16	–	17.3–20	10.7–11.1	16.7–17	–	Rühm (1956)
	Female	547–701	19–20	–	125–163	25–27	10.3–11.7	12.5–15.6	77–78	
*E. piniperdae*	Male	483–750	14–15	–	–	31–44	9.3–12.5	19.4–25	–	Kakulia and Lazarevskaja (1965)
	Female	615–780	14–16	–	–	28–38	10–12	–	67–75	
*E. prolobos*	Male	610–660				35	7	14	–	Massey (1964a, 1964b)
	Female	700–810	12	–	–	35	8.5	–	79	
*E. propora*	Male	630–900	9.8–24	15–22	–	32.6–45.1	–	23.3–40.4	–	Yang (1985)
	Female	900–1,260	15–30.5	–	–	34.5–54	–	–	64.4–82.6	
*E. scolyti*	Male	690–765	15–17	14–15	–	32.8–36.4	6.7–8.4	25.5–27.1	–	Rühm (1956)
	Female	900–1,185	21	–	291–315	32.1–48.4	8.0–17.8	2.8–4.1	65–75	
*E. skrjabini*	Male	370–420	13–14	–	–	28–35	–	13.2–15	–	Lazarevskaya (1961)
	Female	466–508	13–15	–	–	28.5–35.5	8.1–13.7	–	78–81	
*E. stammeri*	Male	796–867	15–17	18–21	–	34.6–41.3	8.9–9.2	16.9–20.2	–	Körner (1954)
	Female	645–931	17–18	–	–	31.9–35.8	7.6–10	12.4–17.2	68–77	
*E. tuerkorum*	Male	645–690	15–18	11–14	–	32.8–36.8	7.1–8.4	23–25.5	–	Rühm (1956)
	Female	705–735	16–19	–	147–158	35–40.2	9.5–9.6	4.5–5.0	77–80	
*E. tenuidens*	Male	750	–	–	–	–	–	–	–	Thorne (1935)
	Female	800	–	–	–	–	–	–	–	
*E. zwoelferi*	Male	682–1,056	21–37	16–24	–	33–35.8	8.6–9.7	17–17.3	–	Rühm (1957)
	Female	1,056–1,320	34–40	–	270–344	33–35	8.7–10.7	13.1–14.9	66–68	
			Species described without males and unknown incisures in lateral field							
*E. amitini*		523	10–14	–	–	27.5	11.5	–	81	Fuchs (1937) and Rühm (1956)
*E.hylastophilus*		893	17	–	–	37.2	21.7	16.8	72.4	Fuchs (1930)
*E. larici*		480–800	17–23		–	24.7–42.7	8.1–9.7	–	–	Lazarevskaya (1963)
*E. olitorius*		480–550	21–22	–	–	28–33	–	15–16	60–80	Chaturvedi and Khera (1977)
*E. typographi*		705–735	23	–	–	24.5–25.2	6.9–7.2	5.1–5.3	80–81	Rühm (1956)
^a^ *E.berbericus*		512–691	19–22	–	–	28.7–36.3	7.2–9.6	16.7–19.2	79.1–81.4	Alvani et al. (2016)

Note: ^a^Females were described, having three lateral lines; ^b^only 1 male was presented by (Kanzaki et al., 2008); ^c^cumulative results of all populations mentioned by Kanzaki et al. (2008).

Based on the similar tail morphology, the new species comes closer to *E. berbericus*, *E. joyceae*, *E. oleae*, *E. ibericus*, and *E. taiwanensis*. It could be differentiated from *E. berbericus* by having excretory pore located at or posterior to the nerve ring vs anterior, hemizonid located immediately posterior to excretory pore vs ca one median bulb length posterior to the excretory pore, shorter stylet length 14 (12.4–16.4) vs 20.5 (19–22) μm and male (present vs absent). From *E. joyceae* by the position of hemizonid (posterior to excretory pore vs anterior), the shape of spermatheca (squarish vs oblonged), female tail terminus without spike vs having lateral spike in 31% of females, male caudal papilla pairs (3 vs 1). From *E. oleae* by lip morphology (smooth, hemispherical vs frontal outer margin of lips expanded, forming a small bowl-like structure), shorter length of PUS=11.3 (7.1–14.7) vs 52 (43–73) μm and shorter spicule length 13.2 (12.7–13.7) vs 16.5 (16–18) μm. From *E. ibericus* by tail terminus shape (finely rounded vs sharply pointed), male (abundant vs rare), and spicules shape (capitulum with slight depression near calomus-rostrum junction vs capitulum without clear depression). From *E. taiwanensis* by number of lateral line (4 vs 3), position of excretory pore (same level of nerve ring vs one body diam. posterior to nerve ring), spicule morphology (condylus rounded to triangular), rostrum with a rounded tip, distal end of spicule smooth vs condylus broadly rounded with slightly dorsal bent, rostrum pointed, distal ends of spicules broadly rounded.

### Molecular profiles and phylogenetic status

**Figure 4:**
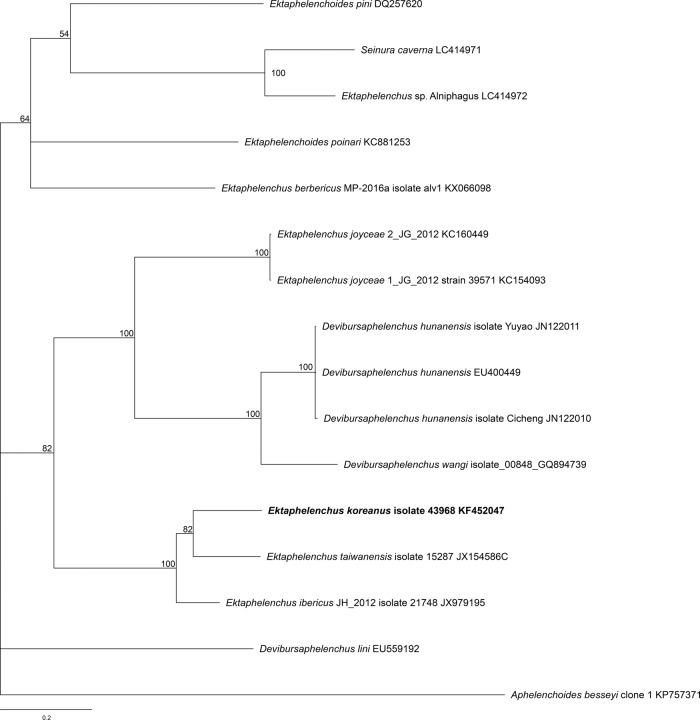
Phylogenetic relationships of *Ektaphelenchus koreanus* n. sp. and aphelenchid nematodes based on full length of ITS. The 100001st Bayesian tree inferred from ITS under TVM+I+G model (ln L = −14,363.6136; freqA = 0.2773; freqC = 0.1724; freqG = 0.2291; freqT = 0.3213; R(a) = 1.0903; R(b) = 3.5149; R(c) = 1.7693; R(d) = 0.9479; R(e) = 3.5149; R(f) = 1.0000; Pinva = 0.0940; Shape = 1.3200). *Aphelenchoides besseyi* served as the outgroup species. Posterior probability values exceeding 50% are given on appropriate clades.

Full-length sequence of the ITS region of rDNA gene was obtained with the accession numbers KF452047 (1,028 bp). The sequences were aligned by MAFFT and modified in a data set of 1,912 characters. Phylogenetic relationships among the isolates for each data set were determined using Bayesian inference (BI), with *Aphelenchoides besseyi* (KP757371) as outgroup. The 50% majority rule consensus phylogenetic trees were generated from ITS data set alignment to BI analysis under the TVM+I+G model (Fig. 4). The new species forms a well-supported molecular clade with two *Ektaphelenchus* species: *E. taiwanensis* (JX154586) and *E. ibericus* (JX979195). And the new species shared the sequence identity values of 77.65 and 80.91% with these two sister species, respectively.

## Discussion

Most of *Ektaphelenchus* species were described from pine trees (Rühm, 1956; Massey, 1974) and associated with insect host (Hunt, 1993), but little is known about their actual biology and mode of interaction with insect vectors or other nematode species (Gu et al., 2013a, 2013b). Previously Gu et al. (2013a) reported the feeding of *E. taiwanensis* on *Aphelenchoides* sp. This new species was also observed feeding on *Aphelenchoides* sp., it is presumed that some *Ektaphelenchus* species may have some predatory role against Aphelenchids or possibly on insects. This aspect of *Ektaphelenchus* species needed more attention and demanded further studies to explore the bio-control potential.

Most of the previously described species are devoid of light microscopy and very few details available for these species. During our literature studies, it is found that *E. chalcographi (* Kurashvili et al., 1980) do not have any comparable illustrations and morphological data that can be considered sufficient to declare the species status, therefore this species is not included in Table 2.

Moreover, several exclusive species differentiating characters were found while studying *Ektaphelenchus* literature such as all of the species were described as having a distinct constriction on the lip region, however, *E. olitorius*, *E. ibericus*, *E. propora*, *E. sandiaensis*, *E. taiwanensis*, and *E. riograndensis* lip region described as without a constriction.

The stylet length and morphology is also an important diagnostic character, majority of *Ektaphlenchus* described having average length of 20 μm or below. However, some species have comparatively longer stylets, i.e. *E. betulae*, *E. dendroctoni*, *E. obtusus*, *E. josephi*, *E. riograndensis*, *E. propora*, *E. scolyti*, and *E. zwoelferi* (see Table 2). The presence/absence of stylet knobs is also a differentiating character, out of 28 valid species only 9 species described having a prominent stylet knobs, i.e. *E. amitini*, *E. betulae*, *E. dendroctoni*, *E. hylastrophilus*, *E. josephi*, *E. scolyti*, *E. piniperdae*, *E. prolobos*, and *E. tenuidens*.

Excretory pore located posterior to nerve ring in majority of species, however, it is described as anterior in *E. betulae*, *E. berbericus*, *E. scolyti*, *E. zwoelferi*, while at the same level of nerve ring in *E. macrobulbosus* and *E. stammeri*. Hemizonid is difficult to observe and this character was not described in several of old description. It is generally located immediately posterior to excretory pore, however, in the descriptions of *E. joyceae*, *E. riograndensis*, and *E. sandiaensis* it is located anterior to excretory pore.

Number of caudal papillae is also an important species differentiating character, majority of *Ektaphelenchus* are described as having three pairs of papillae, two pairs were only described for *E. dendroctoni*, *E. macrobulbosus*, *E. riograndensis*, *E. propora*, *E. tuerkorum* and single pair was described for *E. joyceae*. Single mid ventral pre-anal papillae is difficult to observe but it was only described in *E. goffarti*, *E. stammeri*, *E. olea*, and *E. tenuidens*.

Several of *Ektaphelenchus* species were described without male, hence the female tail terminus morphology is very important diagnostic character. Fortunately, *Ektaphelenchus* species can be categories based on female terminus with broadly rounded terminus (*E. amitini*, *E. larici*, *E. obtusus*, *E. typographi*, *E.skrjabibi*, *E. macrobulbosus*, and *E. sandiaensis*), finely rounded terminus (*E. betulae*, *E. berbericus*, *E. josephi*, *E. propora*, *E. riograndensis*, and *E. zwoelferi*), pointed terminus (*E. dendroctoni*, *E. ibericus*, *E. koreanus* n. sp., *E. taiwanesis*, *E. stammeri*), bluntly pointed terminus (*E. olitorius*, *E. joyceae*, *E. tenuidens*, and *E. tuerkorum*), mucronated terminus (*E. scolyti*) and a mixture of pointed and rounded terminus (*E. olae*, and *E. prolobos*).

This genus receives less attention as none of the *Ektaphelenchus* species was reported to cause potential damage to the pine trees. In addition to this, only recently described species were equipped with molecular characterization. From China, only *E. propora* and *E. macrobulbosus* have been documented (Yang, 1985; Qin and Pan, 2003). Several factors contribute to the low diversity of *Ektaphelenchus* species recorded in Mainland China, such as being devoid of morphological differences, operational and comparable criteria, the unwillingness of nematologists, and impact factors of the journals. These issues have plagued the systematics of invertebrates for decades, and *Ektaphelenchus* have been more prone to this than others.

On the other hand, several *Ektaphelenchus* species were intercepted from imported packaging materials at Ningbo port. This new species was detected in the pine tree sample imported from Korea. To our best knowledge, none of the *Ektaphelenchus* species was ever reported from Korea. It is also noted that the recent detection of *E. joyceae* from Korean and Japanese samples suggests that presumably only a small fraction of the actually existing taxa is known so far. There are still large geographical areas insufficiently studied for their diversity.
